# *Toxoplasma gondii* Inhibits Covalent Modification of Histone H3 at the IL-10 Promoter in Infected Macrophages

**DOI:** 10.1371/journal.pone.0007589

**Published:** 2009-10-27

**Authors:** Jin Leng, Eric Y. Denkers

**Affiliations:** Department of Microbiology and Immunology, College of Veterinary Medicine, Cornell University, Ithaca, New York, United States of America; CNRS/Université de Toulouse, France

## Abstract

Infection of macrophages with the protozoan parasite *Toxoplasma gondii* results in inhibition of a large panel of LPS-responsive cytokines, including TNF-α, while leaving others such as IL-10 intact. Recent studies provide evidence that the parasite interferes with chromatin remodeling at the TNF-α promoter that is normally associated with LPS stimulation, but that is not required for TLR4 induction of IL-10. Here, we examined the effect of *Toxoplasma* on IL-10 induced by simultaneous signaling through TLR4 and FcγR, a combined stimulus that triggers histone H3 covalent modification at the IL-10 promoter resulting in high level IL-10 cytokine production. We show that the parasite inhibits high level IL-10 production and prevents histone H3 Ser^10^ phosphorylation and Lys^9/14^ acetylation at the IL-10 promoter. These results provide compelling evidence that *T. gondii* targets the host cell chromatin remodeling machinery to down-regulate cytokine responses in infected macrophages.

## Introduction

The protozoan *Toxoplasma gondii* is an opportunistic apicomplexan parasite with worldwide distribution in humans and animals. Although normally causing an asymptomatic infection, the parasite can emerge as a dangerous pathogen in immunodeficient hosts [1], [2]. Previous work by us and others has found that macrophages and dendritic cells, important reservoirs of in vivo infection, become nonresponsive to Toll-like receptor (TLR) and IFN-γ receptor activation [3], [4], [5], [6], [7], [8], [9]. Thus, *T. gondii-*infected mouse bone marrow-derived macrophages (BMMØ) are strongly inhibited in their ability to produce a large battery of proinflammatory mediators during stimulation with TLR4 ligand lipopolysaccharide (LPS) [10]. Importantly, not all LPS-responsive genes are suppressed by the parasite. In particular, TLR4 stimulation continues to elicit IL-10 production even when macrophages are infected with tachyzoites [10].

Recently, we conducted a detailed analysis of the activity of the gene encoding TNF-α, a cytokine that is strongly suppressed by *Toxoplasma* [11]. We found that transcription factors associated with the TNF-α promoter, such as NFκB, cAMP-responsive element-binding protein (CREB) and c-Jun, were activated and translocated into the nucleus normally during LPS stimulation of infected cells. Nevertheless, using chromatin immunoprecipitation (ChIP), we obtained evidence that these factors were unable to bind to their target sequences on the native TNF-α promoter after parasite infection [11]. TLR4 triggering of BMMØ resulted in Ser^10^ phosphorylation and Lys^9/14^ acetylation on histone H3 at the TNF-α promoter, epigenetic changes associated with increased transcriptional activity [12]. However, *Toxoplasma* infection prevented these covalent modifications. When we examined the IL-10 promoter, we found that the relatively low level of cytokine produced during LPS stimulation occurred in the absence of histone H3 modification, providing a possible explanation for the lack of suppressive effect of the parasite on this particular cytokine.

Other studies have also indicated that LPS activation of macrophages induces only low amounts of IL-10 and no significant modification of histone H3 [13], [14]. However, combining FcγR ligation with TLR4 stimulation triggers high level IL-10 production, and this is associated with ERK mitogen-activated protein kinase-dependent Ser^10^ phosphorylation and Lys^9/14^ acetylation of histone H3 on the IL-10 promoter. Based upon our findings at the TNF-α promoter [11], it was our prediction that high level IL-10 synthesis stimulated by LPS and immune complex (IC) would be suppressed by the parasite, even though low level LPS-induced IL-10 was not affected. Here, we tested this prediction. We found that, as with the TNF-α promoter during LPS stimulation, *Toxoplasma* blocked histone H3 covalent modification at the IL-10 promoter during stimulation with LPS and IC. These combined data provide strong support for a new model in which *T. gondii* targets histone modification rather than the activity of specific transcription factors, and in this way the parasite silences multiple host genes using a common mechanism of suppression.

## Results

### *Toxoplasma* blocks high level IL-10 production stimulated by combined LPS and immune complex stimulation

**Figure 1:**
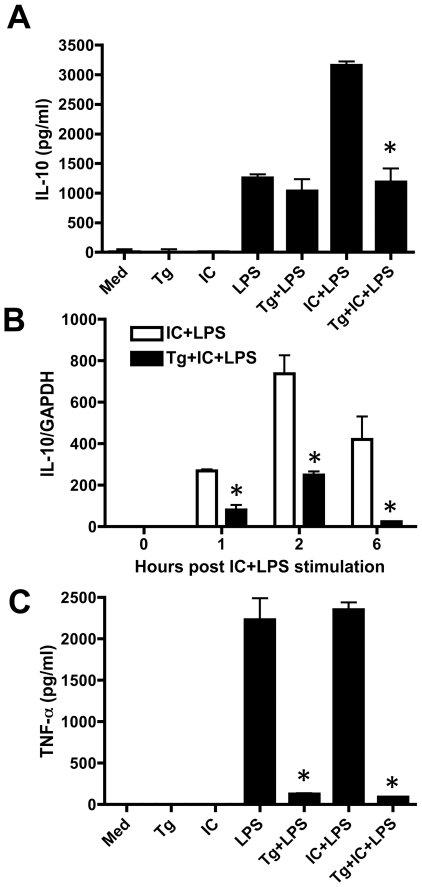
*Toxoplasma* inhibits superinduction of IL-10 stimulated by LPS and IC. Macrophages were infected with *T. gondii* and 18 hr later cells were stimulated with either LPS (10 ng/ml) or LPS plus immune complex. 6 hr later supernatants were collected for IL-10 (A) and TNF-α ELISA (C) and at the indicated time points cells were harvested and RNA was extracted for real-time RT-PCR analysis of IL-10 mRNA levels relative to GAPDH (B). Error bars indicate SD of triplicate samples. Each experiment was repeated a minimum of 3 times. In A and B, * p<0.05 comparing IC+LPS to Tg+IC+LPS. In C, * p<0.05 comparing LPS to Tg+LPS and IC+LPS to Tg+IC+LPS. Med, medium; Tg, *T. gondii*, IC, immune complex.

We previously reported that *Toxoplasma*-infected BMMØ were suppressed in their ability to produce TNF-α after LPS/TLR4 stimulation, but that low level IL-10 production was not affected by the parasite [11]. Here, we asked if high-level IL-10 induced by LPS in combination with IC was blocked by the parasite. As expected, stimulation with LPS alone induced low levels of IL-10, and this response was not affected by *T. gondii* (Fig. 1A). However, BMMØ produced approximately three-fold more IL-10 when the cells were triggered with LPS + IC, although IC alone failed to elicit this cytokine. Notably, this response was down modulated to levels obtained with LPS alone when cells were pre-infected with *Toxoplasma* (Fig. 1A).

We also measured IL-10 mRNA levels following stimulation with the combination of LPS and IC. Previously, we found that the parasite has no effect on increased IL-10 mRNA levels following LPS stimulation [11]. Here, we found an approximately 10-fold increase in IL-10 mRNA after stimulation with LPS + IC relative to triggering with LPS alone (Fig. 1B). Infection of cells with *T. gondii* resulted in suppressed IL-10 mRNA induction following LPS + IC stimulation (Fig. 1B). We conclude that *Toxoplasma* does not block low-level IL-10 induced by LPS, but that high level IL-10 production triggered by the combination of LPS and IC is sensitive to suppression by the parasite.

We examined the effect of LPS + IC on TNF-α levels in BMMØ. In this case, FcγR ligation did not further enhance TNF-α production over levels obtained with LPS alone (Fig. 1C). Unlike the case of IL-10, infection with *T. gondii* inhibited TNF-α production during LPS and LPS + IC stimulation to background levels.

### *T. gondii* inhibits histone H3 covalent modification triggered by LPS + IC

**Figure 2:**
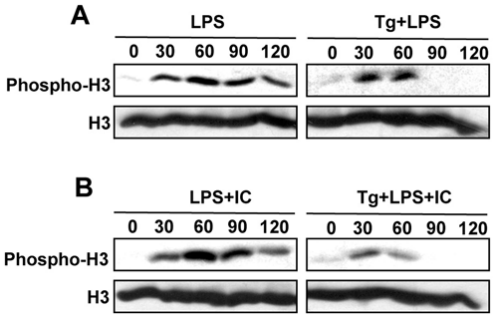
*Toxoplasma* interferes with global Ser^10^ phosphorylation of histone H3 induced by LPS and immune complex. Macrophages were infected with tachyzoites (2∶1 ratio of parasites to cells), then 18 hr later cells were stimulated with LPS (10 ng/ml) (A) or the combination of LPS and immune complex (B). Cells were lysed at the indicated time points and lysates were subjected to Western blot analysis using Ab specific for Ser^10^ phosphorylated histone H3 and total histone H3. Tg, *T. gondii*; IC, immune complex.

We next assessed if global changes in histone H3 during LPS + IC stimulation were affected by *Toxoplasma*. We used Western blot analysis to examine histone H3 Ser^10^ phosphorylation following stimulation of infected cells with LPS and LPS + IC. As reported previously, LPS stimulation induced an increase in Ser^10^ phosphorylation of histone H3 in total macrophage lysates, and this response was inhibited when cells were preinfected with *Toxoplasma* (Fig. 2A). In similar fashion, stimulation with LPS + IC triggered increased Ser^10^ histone H3 phosphorylation, and this response was blocked by *T. gondii* (Fig. 2B). We also examined histone H3 Lys^9/14^ acetylation in cell lysates but found that nonstimulated, noninfected BMMØ possessed constitutively high levels of this histone modification that were not noticeably affected by LPS + IC stimulation (data not shown).

**Figure 3:**
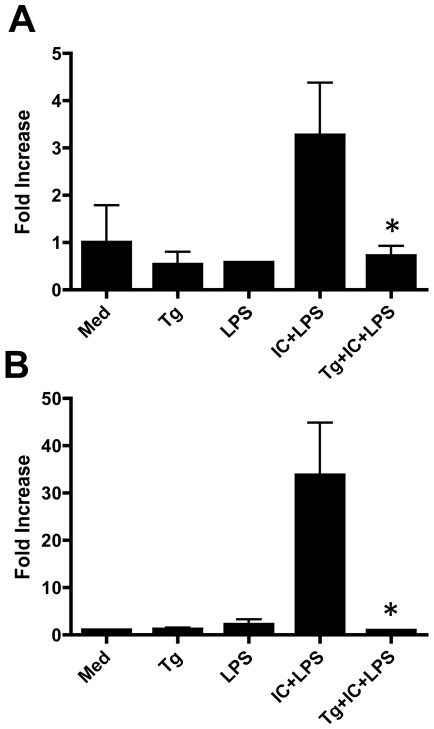
*T. gondii* inhibits LPS + immune complex-inducible histone H3 phosphorylation and acetylation at the IL-10 promoter. Macrophages were infected with tachyzoites at a parasite to cell ratio of 2∶1. After 18 hr incubation cells were subjected to stimulation with LPS alone or LPS in combination with immune complex. At either 30 min (A) or 60 min (B) cells were collected and samples were subjected to ChIP analysis using anti-phospho-histone H3 (Ser^10^) (A) or anti-acetyl-histone H3 (Lys^9/14^) (B). Real-time PCR analysis was carried out using primers specific for the IL-10 promoter. The data were normalized to corresponding input controls. The error bars show SD values for triplicate samples. This experiment is representative of three performed. * p<0.05, comparing IC+LPS to Tg+IC+LPS. Med, medium; Tg, *T. gondii*; IC, immune complex.

Histone modifications detected in whole cell lysates are not necessarily reflective of chromatin status at specific loci. Therefore, we employed chromatin immunoprecipitation (ChIP) to evaluate the effect of *Toxoplasma* on the status of IL-10 promoter-associated histone H3, focusing on Ser^10^ phosphorylation and Lys^9/14^ acetylation in response to LPS + IC. Work by others has established that IL-10 superinduced by combined stimulation with LPS and IC results in chromatin remodeling, allowing access of transcription factors at the IL-10 promoter [13], [14]. We specifically examined modification of histone H3 at the nucleosome 2 position of the IL-10 promoter, since this region is most strongly associated with histone phosphorylation and acetylation following stimulation through TLR4 and FcγR [14]. In accord with previous data, stimulation of BMMØ with LPS alone failed to induce changes in phosphorylation or acetylation of histone H3 at the IL-10 promoter (Fig. 3A and B). However, stimulation with LPS and IC triggered rapid Ser^10^ phosphorylation and Lys^9/14^ acetylation, and both of these responses were suppressed when cells were pre-infected with *Toxoplasma* (Fig. 3A and B). We conclude that histone H3 modification-independent IL-10 induction is not sensitive to suppression by *T. gondii*, but that histone H3 modification-dependent IL-10 synthesis, like control of TNF-α, is blocked by infection.

## Discussion

Superinduction of IL-10 in macrophages requires two signaling cascades. One emanates from TLR4 and results in activation of IL-10 transcription factors such as STAT3 and Sp1 [15]. The second pathway is activated through FcγR and results in chromatin remodeling at the IL-10 promoter to allow access of transcription factors [13], [14]. The results of this study suggest that *Toxoplasma* inhibits the superinduction response by interfering with the chromatin remodeling pathway. These data reinforce recent findings from our laboratory indicating that *T. gondii* has a similar inhibitory effect on LPS-initiated histone H3 modification at the TNF-α promoter [11]. In that case, we found that although transcription factors were activated normally during LPS stimulation, histone H3 phosphorylation and acetylation were blocked by *T. gondii*. Recent studies have found that histone H3 phosphorylation, but not acetylation, is the proximal event to IL-10 gene transcription [14]. Our finding that *Toxoplasma* possesses inhibitory effects on total levels of histone H3 Ser^10^ phosphorylation suggests that this may be the relevant target of suppression.

The IL-10 molecule is an anti-inflammatory mediator that is important in down-modulating proinflammatory responses to avoid pathology. Its relevance during *Toxoplasma* infection was shown in studies employing IL-10 gene deficient mice, because animals infected with this parasite succumb during acute infection in the absence of IL-10. Death is associated with a runaway proinflammatory cytokine response that, despite controlling the parasite, leads to lethal immunopathology during systemic and oral infection [16], [17]. Similarly, IL-10 has an important role in limiting inflammation during toxoplasmic encephalitis [18]. From the perspective of the parasite, IL-10 production is important to keep the host alive to maximize chances of transmission. Nevertheless, the ability of IL-10 to down-modulate activation of innate immune effector cells such as macrophages suggests that overproduction of this cytokine must be avoided. In this case, uncontrolled parasite replication would rapidly lead to host death, minimizing the chances of transmission to a new host. We previously reported that low-level IL-10 induction triggered by TLR4 was not inhibited by *Toxoplasma*. Here, we now demonstrate that macrophages triggered through TLR4 and FcγR produce much higher amounts of IL-10, and that *T. gondii* blocks this superinduction response to levels achieved by LPS stimulation alone. We hypothesize that inhibition of high but not low level IL-10 is a reflection of the parasite's need to ensure appropriate amounts of IL-10 that avoid immunopathology and permit cyst formation, favoring long-term persistence in the host.

In addition to TNF-α and high level IL-10, many other LPS-responsive cytokines and chemokines are down regulated by *Toxoplasma* infection of macrophages [10]. Similar effects occur in LPS-stimulated dendritic cells, where infection blocks upregulation of MHC class II and costimulatory molecules in addition to TNF-α and IL-12 [5]. It is also known that the parasite inhibits the ability of macrophages and fibroblasts to respond to IFN-γ [8], [19]. For the case of fibroblasts, a large family of IFN-γ-responsive genes is inhibited during *T. gondii* infection [20]. The ability of the parasite to simultaneously down-regulate large subsets of genes during activation with stimuli such as LPS and IFN-γ suggests that targeting chromatin remodeling may be the mechanism that mediates these profound effects.

The mechanism used by *Toxoplasma* to interfere with host chromatin remodeling is presently unclear. The parasite is known to inject rhoptry kinases and phosphatases into the host cytoplasm during infection, and although these molecules relocate to the host cell nucleus, whether they are involved in the effects reported here is not presently known [21], [22], [23], [24]. Nevertheless, it is possible that such molecules interfere with host histone kinases and acetylases. Alternatively, it is possible that parasite-derived effector molecules directly dephosphorylate and deacetylate histone H3.

The findings reported in the present manuscript in combination with other recent results from our laboratory together provide strong evidence for a new view of *Toxoplasma* as an intracellular pathogen that targets chromatin modification rather than (or in addition to) targeting specific transcription factors. The finding that proinflammatory TNF-α and anti-inflammatory IL-10 cytokines are both subject to the same type of regulation by *T. gondii* reinforces the concept that interference with inducible histone modification is a common strategy used by the parasite to influence host gene transcription. These combined studies are the first to demonstrate these effects during protozoan infection, but recent data suggest that some bacterial pathogens, such as *Shigella flexneri*, *Listeria monocytogenes*, and *Mycobacterium tuberculosis* adopt similar strategies during infection [25], [26], [27], [28]. Together, these data support an emerging view of host cell chromatin structure as an important target during microbial pathogenesis.

## Materials and Methods

### Ethics Statement

All work with animals received approval from the Cornell University Institutional Animal Care and Use Committee.

### Mice and Parasites

C57BL/6 mice (6–8 wk of age) were purchased from The Jackson Laboratory. The mice were kept under specific pathogen-free conditions at the Transgenic Mouse Facility, Cornell University College of Veterinary Medicine. The facility is overseen by an Institutional Animal Care and Use Committee. The Type I *T. gondii* parasite strain RH was maintained by bi-weekly passage on human foreskin fibroblast monolayers (American Type Tissue Collection) in DMEM supplemented with 1% Bovine Growth Serum (BGS), 100 U/ml penicillin and 0.1 mg/ml streptomycin. Parasite cultures were tested every 6–8 wk using a PCR-based ELISA (Roche Diagnostics).

### Cell culture

Bone marrow cells were flushed from femur and tibia of C57BL/6 mice and cultured in macrophage medium consisting of DMEM supplemented with 10% BGS, 1 mM sodium pyruvate, 0.1 mM nonessential amino acids, 20% supernatant from L929 cells, 100 U/ml penicillin and 0.1 mg/ml streptomycin. The cells were supplemented with fresh macrophage medium on Day 3 after culture initiation. After 5 days of culture, nonadherent cells were removed, adherent monolayers were washed with PBS and cells were harvested by gentle pipeting with ice-cold PBS. Macrophage infection was accomplished by adding tachyzoites to cell cultures followed by brief centrifugation (200 x g, 3 min) to initiate contact between cells and parasites. Immune complex and LPS were added 18 hr after infection. Cells were recovered at varying times depending upon the assay performed.

### Immune complex preparation

IgG-opsonized erythrocytes (E-IgG) were generated by incubating sheep red blood cells (SRBC, Lampire Biological Laboratories) with anti-SRBC IgG (MP Biomedicals) at nonagglutinating titers for 30 min at room temperature while rotating. Opsonized cells were washed once in Hank's Buffered Saline Solution (Invitrogen Life Technologies) and resuspended in macrophage medium. E-IgG were added to cells at a ratio of 10 E-IgG to 1 macrophage.

### Semiquantitative real-time PCR

RNA was isolated from cells using a commercial kit (RNeasy Mini-kit; Qiagen) and cDNA synthesized according to standard protocols. Real-time PCR was performed with a Power SYBR green kit according to the manufacturer's instructions (Applied Biosystems). Amplification was carried out on an Applied Biosystems 7700 Sequence Detector. The sequences of primers used are indicated in Table 1.

**Table 1:** Primers used in this study.

Primer	Sequence (5′ to 3′)
IL-10 forward	CCT GGC TCA GCA CTG CTA T
IL-10 reverse	GCT CTT ATT TTC ACA GGG GAG AA
GAPDH forward	CCT GAA CAG AAC AGC AAT GGC T
GAPDH reverse	GCT TGA CGG TGT CTT TTG CCT
IL-10 nucleosome 2 forward	GCA GAA GTT CAT TCC GAC CA
IL-10 nucleosome 2 reverse	GGC TCC TCC TCC CTC TTC TA

### Cytokine ELISA

IL-10 in culture supernatants was measured using a commercial kit according to the manufacturer's recommendations (eBioscience).

### Immunoblotting

Anti-phospho-histone H3 (Ser^10^; Cell Signaling) and anti-total histone H3 (Cell Signaling) were used for Western blot analysis. Cells (2×10^6^/sample) were lysed in reducing SDS-PAGE sample buffer, and DNA was sheared by forcing samples 3 times through a 27-guage needle. After 3 min at 100°C, samples were separated by 10% SDS-PAGE and proteins were subsequently electrotransferred onto nitrocellulose membranes. The membranes were blocked in 0.1% Tween 20 in Tris-buffered saline, pH 7.6 (TBST) containing 5% nonfat dry milk for 1 hr at room temperature, followed by overnight incubation (4°C) with Ab in 5% BSA in TBST. After washing blots in TBST, Ab binding was detected with a horseradish peroxidase-conjugated secondary anti-rabbit Ab (Jackson Immunoresearch) in TBST containing 5% nonfat dry milk. Following 1 hr incubation, blots were washed in TBST and developed with a chemiluminescence-based detection system (Cell Signaling).

### Chomatin immunoprecipitation (ChIP)

ChIP-grade Ab to phospho-histone H3 (Ser^10^) and acetylated histone H3 (Lys^9/14^) were obtained from Cell Signaling. Assays were performed using the ChIP-IT enzymatic express kit (Active Motif) according to the manufacturer's instructions. Briefly, cells (1.5×10^7^/sample) were fixed in 1% paraformaldehyde at room temperature for 10 min. Fixation was quenched by adding glycine to the mixture. The cells were then collected by scraping in buffer containing PMSF (100 mM). After brief centrifugation, the macrophages were resuspended in cell digestion buffer (Active Motif) and subjected to enzymatic digestion for 10 min at 37°C. The reaction was terminated by addition of 0.5 M EDTA. Ab were added to the sheared chromatin preparations and the mixture was incubated with Protein G magnetic beads (Active Motif) overnight at 4°C. The precipitated DNA-protein-Ab complexes were then washed and the cross linking was reversed by incubation at 65°C for 4 hr. Proteinase K was added to digest protein and DNA was subsequently purified using ethanol extraction, air dried, and redissolved in 100 µl H_2_0. The retrieved DNA was then subjected to real-time RT-PCR using promoter-specific primers.
